# Hand hygiene compliance to five moments in pediatric and neonatal intensive care units

**DOI:** 10.1186/2047-2994-4-S1-P156

**Published:** 2015-06-16

**Authors:** HIG Giamberardino, APO Pacheco, JM Webler, BM Domiciana, JN Malta

**Affiliations:** 1 Epidemiology and Infection Control Department, Hospital Pequeno Principe, Curitiba, Brazil

## Introduction

Hand hygiene compliance is essential to prevent healthcare associated infections. Direct observation is the gold standard to monitor optimal hand hygiene compliance. These subject in paediatric patients has limited data.

## Objectives

The aim of this study was measure healthcare workers (HCWs) hand hygiene compliance using the five moments of hand hygiene.

## Methods

An observational study of HCWs hand hygiene compliance was conducted in a paediatric teaching hospital. The study was set on four PICUs:Neonatal (16-beds), Cardiac (20-beds), Surgery (12-beds) and General (14-beds). The observer was trained with members of research team. The data were collected manually at predefined timetables (09:00-17:00 hours), in period in 2014 from June to August and from October to December. The standardized WHO form was used. All opportunities observed were classified as one of the five moments of hand hygiene. HCWs were classified as:physicians, nurses, technical nurses, physiotherapists, radiology and laboratory technicians.

## Results

A total of 1227 hand hygiene opportunities were observed, with 56.64% (695) compliance. Regarding hand hygiene compliance stratified by HCWs categories, was observed:68.97% (140/203) nurses, 58.12%(451/776) technical nursing,35.98%(25/57) physicians and others 53.57%(45/84) HCWs. The adherence, of all HCWs, in each opportunity of five moments in hand hygiene, according to frequency was: 62.66%(198/316) 1st moment; 56.06%(111/198) 3rd moment; 54.49%(188/345) 4th moment; 54.17%(26/48) 2nd moment and 53.75%(172/320) for the 5th moment. The hand hygiene compliance by PICUs was: 64.85%(345/532) Cardiac PICU; 61.35%(127/207) NICU;54.39% (99/182) General PICU and 39.24%(124/316) Surgical PICU.

## Conclusion

This tool allows the identification of fragile points of the work process in health assistance. And despite the study, occur in the same hospital, we can find very different perceptions and situations. This indicates, how at the same time, it is simple and complex, but feasible with continuous education, feedbacks and good acceptance systems for hand hygiene. Direct systematic observations offers crucial data on compliance, and fulfill the five moments for hand hygiene is still considered a challenge in infection control.

## Disclosure of interest

None declared.

